# The high prevalence of playing-related musculoskeletal disorders (PRMDs) and its associated factors in amateur musicians playing in student orchestras: A cross-sectional study

**DOI:** 10.1371/journal.pone.0191772

**Published:** 2018-02-14

**Authors:** Laura M. Kok, Karlijn A. Groenewegen, Bionka M. A. Huisstede, Rob G. H. H. Nelissen, A. Boni M. Rietveld, Saskia Haitjema

**Affiliations:** 1 Department of Orthopedics, Leiden University Medical Center, Leiden, The Netherlands; 2 Department of Geriatrics, MC Slotervaart, Amsterdam, The Netherlands; 3 Department of Rehabilitation, Physical Therapy Science & Sports, Rudolf Magnus Institute of Neuroscience, University Medical Center Utrecht, Utrecht University, Utrecht, The Netherlands; 4 Medical Center for Dancers and Musicians, Haaglanden Medical Center, The Hague, The Netherlands; University of Sydney, AUSTRALIA

## Abstract

**Objective:**

Despite the high number of amateur musicians in the general population, little is known about the musculoskeletal health of amateur musicians. Playing a musical instrument is supposed to be a risk factor for the development of musculoskeletal complaints. This study aimed to evaluate playing-related musculoskeletal disorders (PRMDs) among amateur musicians playing in student orchestras.

**Design:**

A cross-sectional study.

**Participants:**

357 members of eleven Dutch student orchestras across the Netherlands were included in this study.

**Intervention:**

A paper-based questionnaire on PRMDs was used.

**Outcome measures:**

Sociodemographic characteristics and PRMDs were evaluated using an adaptation of the Nordic Musculoskeletal Questionnaire (NMQ) and the music module of the Disabilities of Shoulder and Hand (DASH) questionnaire.

**Results:**

The year prevalence of PRMDs among amateur musicians was 67.8%. Female gender, younger age, higher BMI and playing a string instrument were independently associated with a higher prevalence of PRMDs. The left shoulder was affected more frequently in violinists and violists, whereas the right hand and wrist were more frequently affected in woodwind instrumentalists. Of the subjects with PRMDs during the last week, the score of the music module of the DASH was 18.8 (6.3–31.2)

**Discussion:**

This study is the first to report on PRMDs and its associated factors in a large group of amateur musicians. The prevalence of PRMDs in amateur musicians is high, however the DASH scores reflect a confined impact of these PRMDs on their functioning as a musician. Preventive measures are needed aiming at reducing PRMDs among amateur musicians.

## Introduction

Playing a musical instrument is a risk factor for the development of musculoskeletal complaints, a phenomenon repeatedly confirmed in professional musicians.[1–5] However, only a minority of the musicians is professional, in the Netherlands an estimated 20.000–25.000 of a total population of 17 million people.[6] Contrary, 18% of the Dutch population, more than 3 million people, consider themselves amateur musicians.[7] Among university students the number of amateur musicians is possibly even higher; a Dutch study indicated that 33% of these university students played an instrument.[3]

The reported prevalences of PRMDs in amateur musicians vary greatly, depending on the study design and the population studied. Prevalences of up to 80% have been reported among amateur musicians.[8–12] These numbers seem to outline the prevalence of musculoskeletal complaints in the open population, for example in the Netherlands a year prevalence of 53.4% is reported in a survey in the open population.[13] However, no study directly comparing prevalences has been performed to our knowledge.[2,14,15] Female gender has been associated with a higher prevalence of PRMDs among amateur musicians.[2,11,12] Playing load is another confirmed risk factor among amateur musicians.[8,9,11] A recent cohort study reported a nearly threefold increase in prevalence following a sudden increase in playing time.[8]

However, literature studying the health of the amateur musician is scarce.[8] Several associated factors for PRMDs in professional musicians have not yet been studied in amateur musicians. Among these factors are instrument type, tobacco and alcohol consumption, exercise, playing experience and warming up, and perceived physical burden.[1,16,17] Also several biomechanical factors possibly influence the occurrence of PRMDs in musicians; asymmetric static playing posture, weight of the instrument and elevation of the arms possibly play a role in the development and maintenance of PRMDs.[14,16,18] Each instrument thereby has its own potential risk factors due to differences in playing technique.[19] Within the general population, additional to the music-specific risk factors, other determinants such as age, comorbidity and physical demands were found to be risk factors for musculoskeletal complaints.[20]

Therefore, this study aimed to explore the extent and prevalence of musculoskeletal health problems among amateur musicians. The first objective was to evaluate the prevalence of playing-related musculoskeletal disorders (PRMDs) in amateur musicians.[21] The second objective was to identify factors associated with a higher risk of PRMDs.

## Materials and methods

### Design

A cross-sectional study was performed among university student amateur musicians. Amateur musicians in this study are defined as all musicians who do not currently study at a music academy or have obtained a music academy degree. The timeframe for inclusion was set between February and May 2015 because we wanted to exclude PRMDs related to an increase in playing load related to the start of the orchestra season (September - October) and upcoming performances (November-December, June-July), as an increase in playing load is a known risk factor for PRMDs in amateur musicians.[8]

### Participants

We approached 17 Dutch student orchestras all across the Netherlands for participation in the study. Two orchestras declined and four orchestras were not able to participate within the desired timeframe. Thus, we visited 11 student orchestras (LMK, KAG, SH, WB) during their weekly rehearsals and invited all the musicians who were present to participate in our study. A student orchestra in the Netherlands is an orchestra, mainly consisting of university students, for whom making music is a leisure activity. In other words, these students generally do not study music. However, some orchestra members attended or attend a music academy (fulltime or part-time professional musical education); they were excluded from participation in this study. A certain playing level is required to play in a Dutch student orchestra, as musicians have to play an audition (play for a committee who decides whether the musician has the desired playing capacities) before admission to the orchestra. In all orchestras a classical, symphonic program is played during the study period. The study protocol was reviewed by the regional ethical committee; (METC Zuid-West Holland, registration number 14–086) who decided the Medical Research Act did not apply. According to the Dutch Code of conduct for the use of data in health research, participants were presented with an opt-out and written informed consent was not collected as data were analyzed anonymously.

### Outcome measures

The paper-based questionnaire used in this study has been described in PLOS in detail by Kok et al.[8] In brief, the questionnaire includes sociodemographic characteristics, such as gender, age and lifestyle habits, and music-related questions including instrument and playing experience. The part of the questionnaire focusing on PRMDs is an adaptation of the Nordic Musculoskeletal Questionnaire (NMQ).[22] We used Zaza’s definition of PRMDs: ‘pain and other symptoms that are chronic, beyond your control, and that interfere with the ability to play your instrument at the usual level’.[21] This definition of PRMDs was explicitly mentioned to the participants. Participants were asked if they had experienced PRMDs during the past week, four weeks, three months and year and to identify the location of these PRMDs using the body map of the NMQ. The body map of the NMQ included the following anatomic localizations: mouth/jaw; neck; shoulder left; shoulder right; upper back; elbow left; elbow right; lower back; hand/wrist left; hand/wrist right; hip/upper leg left; hip/upper left right; knee left; knee right; foot/ankle left; foot/ankle right. To assess the degree of impact on musical activity, the music module of the DASH (Disabilities of Arm, Shoulder and Hand) was included in the questionnaire. This music module of the DASH consists of four questions evaluating the impact of the complaints on the ability to play the instrument during the last seven days (S2 Table). Each item was scored on a 5-point Likert scale; 1 representing the best and 5 the worst score on each question The response scores of each item were summed and transferred to a total score ranging from 0 (no disability) to 100 (completely disabled). The total score was calculated by adding the assigned values (1–5) for each response; divide this number by four, subtract one and multiply this number by 25.[23] In case of a missing value on one or more of the DASH questions, the subject was excluded from the DASH analysis.

The following anatomic regions for PRMDs were distinguished: head, mouth/jaw, neck, upper back, lower back, shoulders (left and right), elbows (left and right), hands/wrists (left and right), hips/thighs (left and right), knees (left and right), and feet/ankles (left and right). Instruments were classified following the traditional subdivisions; the category string instruments included the bowed string instruments violin, viola, cello and double-bass. The category woodwind instruments included the instruments flute, bassoon, clarinet, oboe, and saxophone. In the category brass instruments, the horn, tuba, trumpet, trombone and euphonium were included. Percussion, piano and harp were classified as “other”. All responses were entered anonymously into a database, with a unique identifier for each questionnaire to preserve the link between database and paper. All answers were entered into the database as literally as possible. If a range was given, this was changed, in consensus (KAG, SH, LMK), to the lowest number for data entry.

### Data analysis

Baseline variables were represented as medians and quartiles 1 and 3 for continuous variables and as a number with a percentage for categorical variables. A prevalence was calculated for each anatomic region at each time point. This prevalence was also aggregated as prevalence of any complaint at certain points in time and as prevalence of any complaint at any point in time. Associations between patient characteristics, type of musical instruments, playing characteristics and outcome were explored using logistic regression modelling. Outcome was defined as any PRMD at any point in time. Possible risk factors were selected using literature search and expert knowledge. We considered age, alcohol use, BMI, experience, hand dominance, type of instrument, practice, sex, exercise and doing a warming up. We fitted two models. The first model was corrected for age and sex, as these variables are generally considered clinically relevant. Based on literature, in addition we included alcohol use, BMI, experience, hand dominance, type of instrument, practice, exercise and doing a warming up into the full model. The level of significance was set to 0.05. All analyses were performed using R (version 3.2.2) in the RStudio environment. (version 0.99.463)

## Results

The questionnaires were completed by 383 participants from 11 student orchestras across the Netherlands. After exclusion of 26 conservatory students, who were not considered amateur musicians, data of 357 participants were included in the analysis. The participants (28.9% male) were on average 22.4 years old (range 15.5–80.8). All baseline characteristics can be found in Table 1. Most participants played a string instrument (52.1%). A baseline table divided by instrument group can be found in S1 Table. The majority of the string instrumentalists and woodwinds were female (79.6% and 77.1% respectively), while the majority of the brass and other instrumentalists were male (64.4% and 62.5%, respectively).

**Table 1:** Baseline characteristics of the amateur musicians included in this study (n = 357).

Age (years)		22.4 (20.6–24.7)
Sport (hours/week)		2.0 (1.0–3.0)
Alcohol (units/week)		4.0 (2.0–7.0)
BMI (kg/m2)		21.5 (20.0–23.2)
Instrument experience (years)		13.0 (10.0–16.0)
Practice (hours/week)		5.0 (3.8–7.0)
Sex	Female	248 (69.5)
	Male	103 (28.9)
	Missing	6 (1.7)
Smoking	No	318 (89.1)
	Yes	37 (10.4)
	Missing	2 (0.6)
Hand dominance	Right-handed	309 (86.6)
	Left-handed	47 (13.2)
	Missing	1 (0.3)
Warming up	No	189 (52.9)
	Yes	165 (46.2)
	Missing	3 (0.84)
Warming up duration (minutes)		5.0 (5.0–10.0)
Instrument group	String	186 (52.1)
	Woodwind	96 (26.9)
	Brass	59 (16.5)
	Other	16 (4.5)

Numbers are medians with (Q1-Q3) for continuous variables, and numbers with percentages for categorical variables.

### Prevalence of PRMDs

The prevalence of PRMDs in this population was 26.9% in the past week, 33.6% in the past four weeks, 37.3% in the past three months and 67.8% in the last year. String instrumentalists reported the highest number of PRMDs, and the year prevalence in this group was 74.2%. The prevalences of each instrument group are presented in Table 2.

**Table 2:** Prevalence of PRMDs in amateur musicians by instrument group (n = 357).

	Total (n = 357)	String (n = 186)	Woodwind (n = 96)	Brass (n = 59)	Other (n = 16)
One week prevalence	26.9%	32.8%	21.9%	20.3%	12.5%
Four-week prevalence	33.6%	36.6%	33.3%	28.8%	18.8%
Three months’ prevalence	37.3%	41.9%	36.5%	28.8%	18.8%
One year prevalence	67.8%	74.2%	63.5%	57.6%	56.2%

### DASH

Of the subjects with PRMDs during the last week, 94 out 96 subjects completed all questions of the DASH. The score of the music module of the DASH was 18.8 (6.3–31.2) (median and interquartile range). String instrumentalists and instrumentalists in the group ‘other’ with PRMDs during the last week reported the highest DASH scores (18.9 (6.3–34.2) and 25.0 (15.6–34.3) respectively). The results of the individual questions of the music module of the DASH are displayed in S2 Table.

### Location of PRMDs

S1–S3 Figs show the body distribution of PRMDs in the different instrument groups. Table 3 presents the corresponding year prevalences for each body region in each instrument group are shown. Of the string instrumentalists, 52.2% reported left shoulder PRMDs during the past year. In this group, 59.4% of the violinists and violists and 36.2% of the cellists and double-bass players reported left shoulder PRMDs during the last year. Also, neck and back problems were reported more frequently among string instrumentalists. Among woodwind instrumentalists the right hand was more often affected than the left (24.0% versus 7.3%). This difference was found in all the instrumental groups of the woodwind section.

**Table 3:** Distribution of PRMDs over various instrumental groups (one-year prevalence).

	Strings (n = 186)	Woodwind (n = 96)	Brass (n = 59)	Other (n = 16)
Head	6 (3.2%)	4 (4.2%)	2 (3.4%)	0
Mouth / Jaw	20 (10.8%)	20 (20.8%)	12 (20.3%)	0
Neck	69 (37.1%)	24 (25.0%)	9 (15.3%)	2 (12.5%)
Shoulder(s)	110 (59.1%)	35 (36.5%)	17 (28.8%)	7 (43.8%)
Shoulder left	97 (52.2%)	24 (25.0%)	12 (20.3%)	5 (31.2%)
Shoulder right	61 (32.8%)	29 (30.2%)	13 (22.0%)	5 (31.2%)
Upper back	60 (32.3%)	15 (15.6%)	6 (10.2%)	1 (6.2%)
Lower back	44 (23.7%)	12 (12.5%)	9 (15.3%)	2 (12.5%)
Elbow(s)	5 (2.7%)	2 (2.1%)	1 (1.7%)	1 (6.2%)
Elbow left	4 (2.2%)	0	1 (1.7%)	1 (6.2%)
Elbow right	2 (1.1%)	2 (2.1%)	1 (1.7%)	0
Hand(s) / Wrist(s)	49 (26.3%)	25 (26.0%)	6 (10.2%)	4 (25.0%)
Hand / Wrist left	34 (18.3%)	7 (7.3%)	4 (6.8%)	2 (12.5%)
Hand / Wrist right	29 (15.6%)	23 (24.0%)	4 (6.8%)	3 (18.8%)
Hip(s) / Upper leg(s)	2 (1.1%)	1 (1.0%)	0	0
Hip / Upper leg left	2 (1.1%)	0	0	0
Hip / Upper leg right	1 (0.5%)	1 (1.0%)	0	0
Knee(s)	3 (1.6%)	0	0	0
Knee left	2 (1.1%)	0	0	0
Knee right	1 (0.5%)	0	0	0
Foot / Feet / Ankle(s)	0	0	0	0
Foot / Ankle left	0	0	0	0
Foot / Ankle right	0	0	0	0

### Risk factors for PRMDs

Our logistic regression model showed that younger age (OR 0.94 (0.90–0.97)), higher BMI (OR 1.10 (1.00–1.21)) and female sex (OR 2.90 (1.78–4.77)) were independently associated with a higher prevalence of PRMDs. The age effect remained present after exclusion of participants aged 35 and older (n = 12) (OR 0.84 (0.77–0.92)). Table 4 shows the results of the sex- and age-corrected model, as well as the fully adjusted model.

**Table 4:** PRMDs in amateur musicians (n = 357); logistic regression modelling.

	Model adjusted for age and gender OR (95% CI)	Full model OR (95% CI)
Age	0.94 (0.90–0.97)	0.93 (0.88–0.98)
Alcohol	0.99 (0.94–1.04)	0.97 (0.92–1.03)
BMI	1.10 (1.00–1.21)	1.14 (1.03–1.27)
Hand dominance (right-handedness)	0.84 (0.41–1.66)	0.78 (0.35–1.64)
Playing experience	0.99 (0.94–1.04)	0.97 (0.92–1.03)
Brass instrument (vs string)	0.69 (0.35–1.39)	0.54 (0.24–1.22)
Other instrument (vs string)	0.79 (0.26–2.63)	0.65 (0.20–2.28)
Woodwind instrument (vs string)	0.54 (0.31–0.96)	0.37 (0.20–0.70)
Practice (weekly playing load)	0.96 (0.90–1.04)	0.92 (0.85–1.00)
Female sex	2.90 (1.78–4.77)	2.28 (1.25–4.17)
Sport	1.01 (0.91–1.13)	1.02 (0.91–1.15)
Warming up (No)	0.95 (0.59–1.53)	0.71 (0.39–1.26)

Numbers are odds ratios (95% CI)

Hand dominance was not significantly associated in our regression model with the prevalence of PRMDs in the complete group of musicians. However, left-handed brass instrumentalists reported a higher number of PRMDs than their right-handed colleagues. The prevalences related to hand dominance are presented in the S3 Table.

## Discussion

This study aimed to explore the extent and prevalence of the musculoskeletal health problems among amateur musicians. The year prevalence of PRMDs among amateur musicians in this study was 67.8%. Female sex, younger age, higher BMI and instrument group were independently associated with a higher prevalence of PRMDs. The left shoulder was affected frequently among violinists and violists, whereas the right hand and wrist were frequently affected in woodwind instrumentalists. This study is the first in the literature reporting on musculoskeletal health in a large group of amateur musicians. The reported prevalences in this study are in line with the scarce literature about PRMDs among amateur musicians.[8–11] Moreover, our prevalence PRMDs in amateur musicians are very comparable to the prevalences of PRMDs in high-level amateur musicians playing in two renowned Dutch national student orchestras, compared to our study population playing in local student orchestras.[8] Furthermore, the results of our study suggest that playing experience does not influence the occurrence of PRMDs in student amateur musicians.

For this study we choose to evaluate PRMDs by using an adapted version of the NMQ. This has several reasons; at first because it eases comparison to other studies evaluating PRMDs in musicians, as most studies assessing musculoskeletal health of musicians evaluate PRMDs instead of all musculoskeletal complaints.[1,8,11,16,24–26] By using the NMQ clarifying body diagrams are used; above this questionnaire is validated. We did however not revalidate our adapted version as only minor changes were made to the original questionnaire. By using the music module of the DASH we were able to evaluate the impact of the PRMDs, a valuable addition to the prevalence data.

The year prevalence of PRMDs in amateur musicians is comparable to the year-prevalence of PRMDs in professional musicians, as reported in a recent review (41–93%)[1] Also the other prevalences (e.g. week, month, 3-months) are comparable to the prevalence rates of professional musicians, although it should be mentioned that the range of these prevalences in the review is broad.[1] In this study, female sex and instrument group are independently associated with PRMDs among amateur musicians. These gender differences are in line with the literature on professional musicians, in which female musicians report more PRMDs.[1,2] Also the anatomic distribution of PRMDs is comparable to professional musicians.[1] However, when comparing literature on amateur and professional musicians, one should realize that study protocols and definitions of complaints are heterogenous.[1]

The results of our study suggest that playing experience does not influence the occurrence of PRMDs in student amateur musicians. Although a sudden increase of playing load influences the occurrence of PRMDs[8], average playing load does not seem to be related to PRMDs, a finding consistent with the literature on amateur musicians.[14] Practicing more therefore does not seem to reduce or increase PRMDs; there are many possible other variables however which can confound this outcome, for example technical playing level and playing capabilities and difficulty of the played repertoire.

A surprising and significant finding in this study is the higher prevalence of PRMDs at a younger age. This finding cannot be explained by less playing experience, for which we also corrected in our statistical analyses. Furthermore, this effect did not disappear when we excluded participants aged 35 and older from the analysis. A possible explanation for this age dependent difference in this study could be the change in health behavior in the younger students[27,28], which potentially could influence PRMDs. Healthcare providers therefore should be aware of the high prevalence of PRMDs in this younger population. As the effect of age is, however, minor (OR 0.94), future research should re-evaluate whether PRMDs actually are age-dependent.

Another remarkable finding in this study is the higher prevalence of PRMDs in left-handed brass instrumentalists compared to their right-handed colleagues. Although there are only eight left-handed brass instrumentalists included in this study, it is a striking difference between the two groups. Some brass instruments, for example the French Horn, are mainly played left-handed, which could potentially influence this difference. However, other studies evaluating the effect of handedness did not show differences between right- and lefthanded musicians.[29,30] Therefore, future studies among brass instrumentalists could be conducted aiming to clarify this issue of handedness.

As up to 20% of the general population consider themselves amateur musicians[7], PRMDs in this specific group have meaningful consequences. First, these PRMDs may interfere with other activities in daily life, such as work, and thus may have financial consequences, even though making music is not the source of income for amateur musicians. Second, PRMDs can affect the amount of pleasure that playing an instrument generally conveys to the musician, thereby counteracting the mental health benefits. It is therefore surprising that literature on PRMDs in amateur musicians is so scarce. When we compare research on musculoskeletal complaints due to playing a musical instrument with research on those due to sports activities at the amateur level, there are considerable differences, mainly related to knowledge of the origin and treatment of these musculoskeletal complaints.[31–33] The field of performing arts medicine is clearly underdeveloped compared to sports medicine, which comprises extensive research not only on prevalences and risk factors, but also on preventive measures to reduce the number of PRMDs, both in professionals and in amateurs.[34]

No norm scores are available for using the DASH music module to reflect the impact and significance of PRMDs in musicians while playing a musical instrument. Moreover, for the optional modules of the DASH the minimally clinically relevant differences are unknown. The results of the music module of the DASH are comparable with these results in a cohort of high-level amateur musicians playing in national orchestras, in which a DASH score (music module) of 14 is reported.[8] This DASH results therefore implicate that the PRMDs of the subjects do influence their playing behaviour, however, the impact of these PRMDs is confined. The relatively low DASH scores suggest that the severity of the evaluated PRMDs was generally limited.

One of the limitations in this study is the possible selection bias. In general, participants who experience PRMDs are more willing to complete a health-related questionnaire. To prevent selection bias as much as possible, the researchers who visited the orchestras explained the aims of the study and emphasized the importance of completing the questionnaire, regardless of the presence of PRMDs.

Overall, the current study analyses a specific group of amateur musicians. Amateur musicians may comprise a wide range of age groups, as well as different musical activities. There is a clear difference in playing technique and playing habits between musical styles (for example classical versus pop music). In addition, the playing time and experience of amateur musicians vary greatly, which was reflected in our study population. The current study was performed in young amateur instrumentalists, the majority of whom had passed an audition before joining their orchestra, implicating a minimum required level of playing. Thus, our data cannot be extrapolated to all amateur musicians, who may not possess the desired skills to pass such an audition. In addition, vocalists were not included in this study, as we aimed to evaluate the occurrence of PRMDs among instrumentalist musicians. A second limitation concerns the evaluation of sports. As we did not evaluate its intensity or exact physical activity, the reported activities might not be comparable, thereby influencing the regression analysis. Another limitation of this study concerns the musicians who play more than one instrument. In the present study, we chose to analyze data regarding the main instrument, as indicated by the musician. In theory, the PRMDs they reported could have been related to their second instrument. However, for all participants who indicated that they played two instruments, these instruments were within the same instrument category, therefore the effect on our results was most probably small. The musicians in this study were grouped according to posture and instrument. The grouping of instruments is another possible limitation as each group constitutes of different instruments, with differences in playing posture and technique, and therefore differences in musculoskeletal load. However, separately reporting prevalences for each instrument would not be reliable due to the relatively small sizes of these groups. The cross-sectional design of the study prohibits any conclusions regarding the causality of the observed risk factors that were associated with increased PRMDs. Moreover, as we studied amateur musicians, their PRMDs could also have originated from other activities in their daily lives. As most of them were students, for example excessive use of computers or reading textbooks in a wrong posture could have resulted in musculoskeletal complaints that interfere with playing their musical instrument. Our definition of PRMDs aimed to catch all musculoskeletal complaints that interfere with playing, and as such it does not state the source of the complaints.

One of the major strengths of this study is that it reports on the largest population of amateur musicians in literature. Due to this large study group, information on the prevalence of PRMDs and associated factors could be assessed relatively reliably. Moreover, this study was the first to systematically evaluate a collection of potential risk factors among amateur musicians. As the literature on amateur musicians is scarce, the current study fills a knowledge gap in medical science regarding musculoskeletal problems in the general population.

Future research among amateur musicians should aim to evaluate the occurrence and risk factors of PRMDs in other groups of amateur musicians. For example, older amateur musicians and non-classical musicians could be evaluated. This knowledge could serve as a guide for developing suitable preventive measures, for example physical training and educational programs, to prevent the development, longer duration and severity of PRMDs in musicians.

Summarizing, in this study among a large group of amateur musicians playing in student orchestras, 67.8% of the instrumentalists reported PRMDs during the past year. The occurrence of these PRMDs was associated with female sex, younger age, higher BMI and playing a string instrument.

## Supporting information

S1 Table (DOCX)

S2 Table (DOCX)

S3 Table (DOCX)

S1 Dataset (XLSX)

S1 Fig (TIF)

S2 Fig (TIF)

S3 Fig (TIF)
