# Supplementary material for: The high prevalence of playing-related musculoskeletal disorders (PRMDs) and its associated factors in amateur musicians playing in student orchestras: A cross-sectional study
Source: PLoS One. 2018 Feb 14;13(2):e0191772. doi: 10.1371/journal.pone.0191772 (PMC5812604; doi:10.1371/journal.pone.0191772)
Supplement: S1 Table — (DOCX) [file pone.0191772.s001.docx]

***S1 Table: Musicians’ characteristics by instrument group (overall study population n=357)***

|  |  | Strings (n=186) | Woodwind (n=96) | Brass (n=59) | Other (n=16) |
| --- | --- | --- | --- | --- | --- |
| Age (years) |  | 22.4 (20.7-24.6) | 22.1 (20.3-24.3) | 22.7 (20.8-25.2) | 22.4 (20.8-25.8) |
| Sport (hours/week) |  | 2.0 (0.5-3.0) | 2.0 (1.0-3.0) | 1.2 (1.0-2.8) | 1.2 (0.0-2.0) |
| Alcohol (units/week) |  | 3.0 (1.0-6.0) | 3.0 (1.8-5.0) | 6.0 (3.0-10.0) | 10.0 (4.0-15.2) |
| BMI (kg/m2) |  | 21.2 (19.8-23.1) | 21.8 (20.1-23.4) | 21.6 (20.1-23.4) | 22.0 (19.6-24.1) |
| Instrument experience (years) |  | 14.0 (12.0-16.0) | 12.0 (9.8-15.0) | 11.0 (9.0-15.0) | 13.0 (9.8-18.0) |
| Practice (hours/week) |  | 5.0 (4.0-8.0) | 4.0 (3.0-5.2) | 5.0 (3.0-7.0) | 4.0 (3.0-5.0%) |
| Sex | Male | 34 (18.3%) | 21 (21.9%) | 38 (64.4%) | 10 (62.5%) |
|  | Female | 148 (79.6%) | 74 (77.1%) | 21 (35.6%) | 5 (31.3%) |
|  | Missing | 4 (2.2%) | 1 (1.0%) | 0 | 1 (6.3%) |
| Smoking | No | 158 (85.0%) | 86 (89.6%) | 58 (98.3%) | 16 (100.0%) |
|  | Yes | 26 (14.0%) | 10 (10.4%) | 1 (1.7%) | 0 |
|  | Missing | 2 (1.1%) | 0 | 0 | 0 |
| Hand dominance | Right-handed | 164 (88.2%) | 82 (85.4%) | 51 (86.4%) | 12 (75.0%) |
|  | Left-handed | 21 (11.3%) | 14 (14.6%) | 8 (13.6%) | 4 (25.0%) |
|  | Missing | 1 (0.5%) | 0 | 0 | 0 |
| Warming up | No | 62 (33.3%) | 48 (50.0%) | 53 (89.8%) | 2 (12.5%) |
|  | Yes | 122 (65.6%) | 47 (40.0%) | 6 (10.2%) | 14 (87.5%) |
|  | Missing | 2 (1.1%) | 1 (1.0%) | 0 | 0 |
|  |  |  |  |  |  |
| Warming up duration (minutes) |  | 10.0 (5.0-10.0) | 5.0 (2.0-10.0) | 5.0 (3.5-10.0) | 10.0 (10.0-10.0) |
| Instruments |  | Violin 94 (50.5%) | Flute 26 (27.1%) | Horn 20 (33.9%) | Harp 1 (6.3%) |
|  |  | Viola 34 (18.3%) | Oboe 9 (9.4%) | Trombone 30 (50.9%) | Piano 1 (6.3%) |
|  |  | Cello 48 (25.8%) | Clarinet 28 (29.2%) | Bass trombone 3 (5.1%) | Timpani 1 (6.3%) |
|  |  | Double bass 10 (5.4%) | Bass clarinet 2 (2.1%) | Tuba 4 (6.8%) | Percussion 13 (81.3%) |
|  |  |  | Bassoon 9 (9.4%) | Euphonium 2 (3.4%) |  |
|  |  |  | Alto saxophone 17.7 (9.4%) |  |  |
|  |  |  | Baritone saxophone 1 (1.0%) |  |  |
|  |  |  | Tenor saxophone 4 (4.2%) |  |  |

*Numbers are medians with (Q1-Q3) for continuous variables, and numbers with percentages for categorical variables*
