# Supplementary material for: The high prevalence of playing-related musculoskeletal disorders (PRMDs) and its associated factors in amateur musicians playing in student orchestras: A cross-sectional study
Source: PLoS One. 2018 Feb 14;13(2):e0191772. doi: 10.1371/journal.pone.0191772 (PMC5812604; doi:10.1371/journal.pone.0191772)
Supplement: S2 Table — (DOCX) [file pone.0191772.s002.docx]

***S2 Table: DASH performing arts module; results per individual question of subjects with complaints during the last week) (n=94)***

|  | 1 (n) | 2 (n) | 3 (n) | 4 (n) | 5 (n) |
| --- | --- | --- | --- | --- | --- |
| Did you have any difficulty using your usual technique for playing your instrument? | 59 | 26 | 9 | 0 | 0 |
| Did you have any difficulty playing your musical instrument because of arm, shoulder or hand pain? | 25 | 45 | 21 | 3 | 0 |
| Did you have any difficulty playing your musical instrument as well as you would like? | 38 | 36 | 15 | 4 | 1 |
| Did you have any difficulty spending your usual amount of time practicing or playing your instrument? | 43 | 20 | 18 | 12 | 1 |

Score of the performing arts module on 5-point Likert scale; 1 representing the best and 5 the worst score on each question
