# Supplementary material for: The high prevalence of playing-related musculoskeletal disorders (PRMDs) and its associated factors in amateur musicians playing in student orchestras: A cross-sectional study
Source: PLoS One. 2018 Feb 14;13(2):e0191772. doi: 10.1371/journal.pone.0191772 (PMC5812604; doi:10.1371/journal.pone.0191772)
Supplement: S3 Table — (DOCX) [file pone.0191772.s003.docx]

***S3 Table: Prevalence of PRMDs in each instrument group by hand dominance***

|  |  |  |  |  |  |  |  |  |  |  |
| --- | --- | --- | --- | --- | --- | --- | --- | --- | --- | --- |
|  | Strings right-handed (n=164) | Strings left-handed (n=21) | Wind right-handed (n=82) | Wind left-handed (n=14) | Brass right-handed (n=51) | Brass_  left-handed (n=8) | Other right-handed (n=12) | Other left-handed (n=4) |  |  |
| one-week prevalence | 32.9% | 28.6% | 19.5% | 35.7% | 15.7% | 50.0% | 16.7% | 0.0% |  |  |
| 4-weeks prevalence | 36.0% | 38.1% | 32.9% | 35.7% | 27.5% | 37.5% | 25.0% | 0.0% |  |  |
| 3-months prevalence | 42.1% | 38.1% | 39.0% | 21.4% | 25.5% | 50.0% | 16.7% | 25.0% |  |  |
| one-year prevalence | 73.8% | 76.2% | 64.6% | 57.1% | 54.9% | 75.0% | 58.3% | 50.0% |  |  |
|  |  |  |  |  |  |  |  |  |  |  |
