# Supplementary figures and images for: The high prevalence of playing-related musculoskeletal disorders (PRMDs) and its associated factors in amateur musicians playing in student orchestras: A cross-sectional study
Source: PLoS One. 2018 Feb 14;13(2):e0191772. doi: 10.1371/journal.pone.0191772 (PMC5812604; doi:10.1371/journal.pone.0191772)

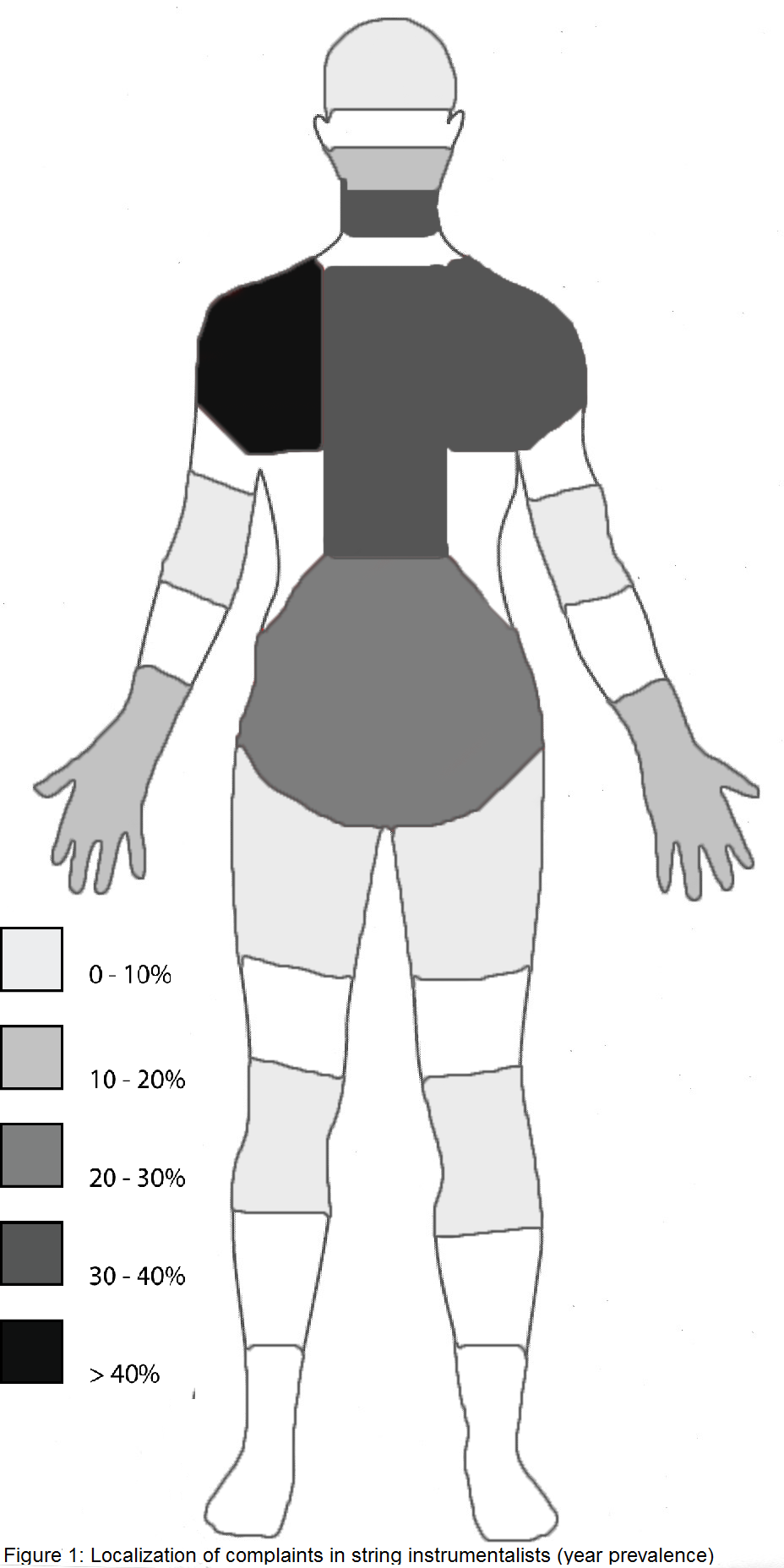

Supplement: S1 Fig — (TIF) [file pone.0191772.s005.tif]

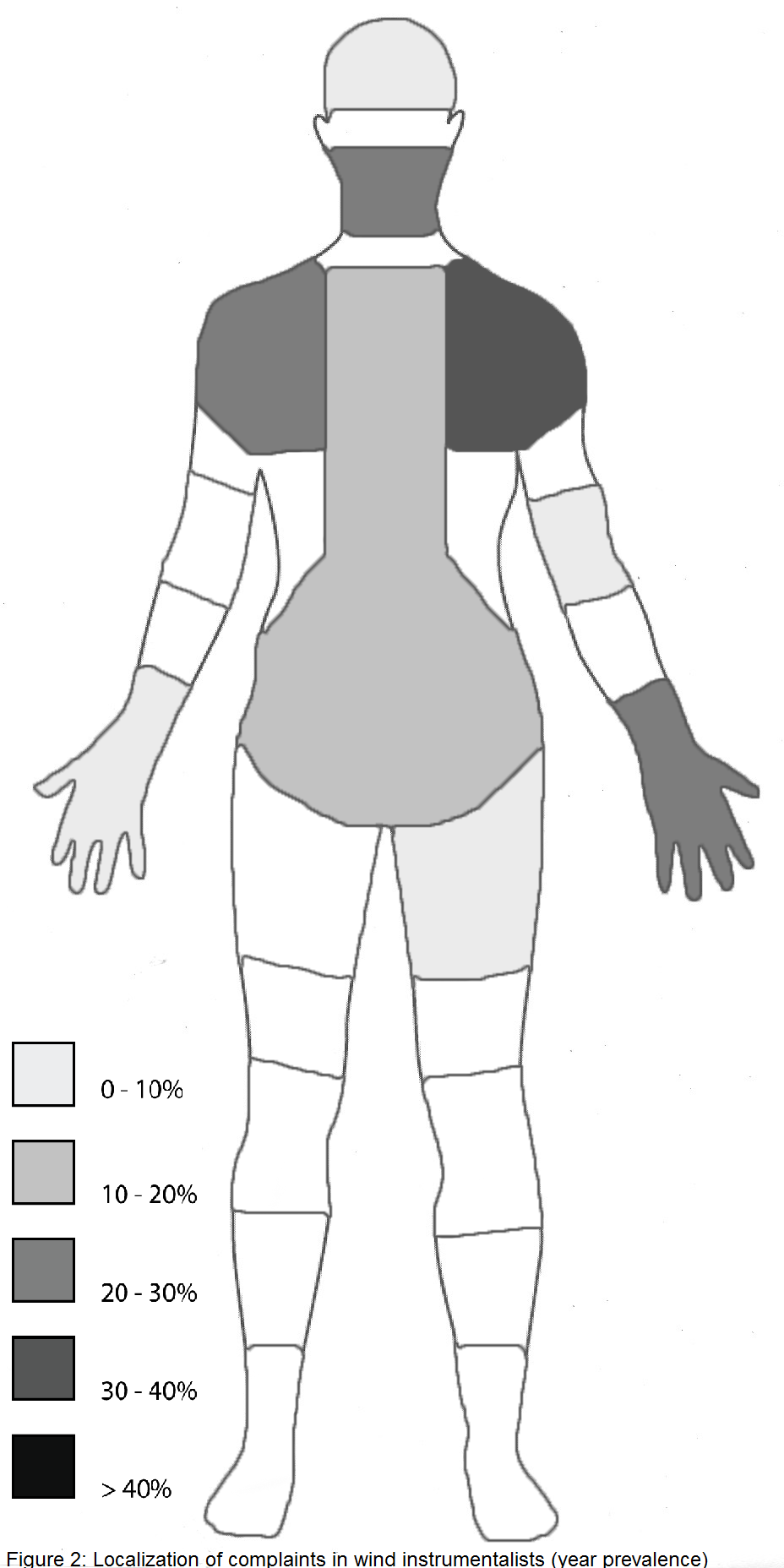

Supplement: S2 Fig — (TIF) [file pone.0191772.s006.tif]

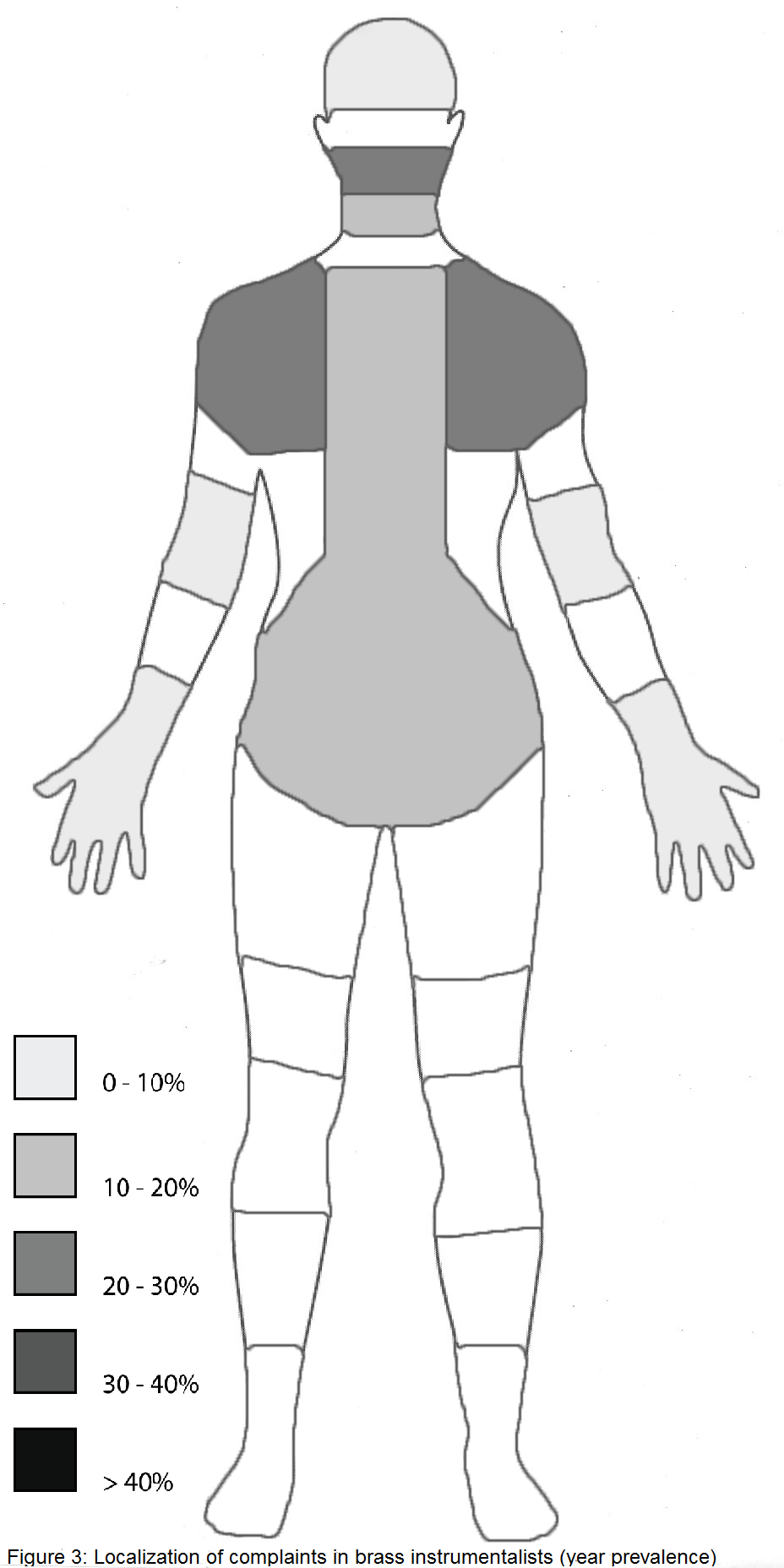

Supplement: S3 Fig — (TIF) [file pone.0191772.s007.tif]
